# Geographic and Socioeconomic Inequalities in Delays in COVID-19 Vaccinations: A Cross-Sectional Study in Indonesia

**DOI:** 10.3390/vaccines10111857

**Published:** 2022-11-02

**Authors:** Hario Megatsari, Dian Kusuma, Ernawaty Ernawaty, Nuzulul K. Putri

**Affiliations:** 1 Department of Health Promotion and Behavior Sciences, Faculty of Public Health, Universitas Airlangga, Surabaya 60115, Indonesia; 2 Department of Health Services Research and Management, School of Health & Psychological Sciences, City University of London, London EC1V 0HB, UK; 3 Centre for Health Economics & Policy Innovation, Imperial College Business School, South Kensington Campus, Exhibition Rd, London SW7 2AZ, UK; 4 Department of Health Policy and Administration, Faculty of Public Health, Universitas Airlangga, Surabaya 60115, Indonesia; 5 Airlangga Centre for Health Policy (ACeHAP), Universitas Airlangga, Surabaya 60115, Indonesia

**Keywords:** COVID-19, vaccine, geographic, socioeconomic, inequality, SDGs

## Abstract

Background: Previous studies have provided evidence of inequalities in the coverage of COVID-19 vaccination. However, evidence of such inequalities in delays in vaccinations is lacking. Our study examined the socioeconomic and geographic disparities in terms of days to get the first and second dose of COVID-19 vaccinations in Indonesia. Methods: We conducted a cross-sectional study using the WhatsApp messaging app and social media platforms during December 2021–February 2022. We distributed the questionnaire through our university network to reach all regions. We included 3592 adults aged 15+ years in our analysis. We used two main dependent variables: days to receive the first dose (after national vaccine rollout) and days to receive the second dose (after receiving the first dose). We examined a range of socioeconomic and geographic indicators, including education level, income level, formal employment, working in health facilities, being a health worker, and region. We controlled for sex, age, religion, and urbanicity. We performed multivariate logistic regressions in STATA 15. Results: Our findings show considerable delays in getting the first dose among participants (160.7 days or about 5.4 months on average) from Indonesia’s national COVID-19 vaccination rollout on 13 January 2021. However, we found a shorter period to receive the second dose after receiving the first dose (41.1 days on average). Moreover, we found significant socioeconomic (i.e., education, income, formal employment, working in health facilities, and being a health worker) and geographic (i.e., in and out of the Java region) inequalities in terms of delays in getting the first dose. However, we did not find significant inequalities in getting the second dose for most inequality indicators, except for working in health facilities. By region, we found that participants living in more deprived areas (out of the Java region) received the second dose 4.9 days earlier. One of the study’s key limitations is that there may be an inherent bias with respect to socioeconomics factors since it was conducted online (web-based). Conclusions: While there were considerable delays in getting the first dose, especially among those of a lower socioeconomic status and those in more deprived areas, the waiting time for the second dose was relatively similar for everyone once they were in the system. Effective efforts to address inequalities are essential to ensuring the effectiveness of the national COVID-19 vaccination rollout.

## 1. Background

The World Health Organization declared COVID-19 as a pandemic more than two years ago, on 11 March 2020. Since then, there have been over 507 million confirmed cases and over 6.2 million deaths reported globally by 25 April 2022 [1]. The pandemic has disrupted people’s day-to-day lives, the economy, and domestic and international travel. Many countries were forced to apply extreme measures by restricting people’s movement (lockdowns) to reduce community transmissions. The pandemic has pressured healthcare services, especially hospitals with an overcapacity of COVID-19 patients and public health systems with massive testing, contact tracing, and other preventative measures [2,3].

The global race to create COVID-19 vaccines that was initiated and fast-tracked throughout 2020 was soon successful in obtaining approvals in some countries. Less than ten months into the pandemic, the United Kingdom started the COVID-19 vaccine campaign in December 2020 using the Pfizer vaccine and in January 2021 using the AstraZeneca vaccine [4,5]. Since then, many countries began the race to fully vaccinate (including the first and second doses of the Pfizer or AstraZeneca vaccines) their entire population, starting with the population groups with the highest risk of infection, such as health workers, and those with the highest risk of dying, such as elderly and immunocompromised residents [6].

Clinical trials have shown that COVID-19 vaccines are effective in protecting individuals, and real-world population studies have shown that COVID-19 vaccines protect against death and severe illness and even reduce disease transmission [7,8]. However, there are socioeconomic and geographic inequalities in the coverage of COVID-19 vaccination between countries, which may reduce vaccine effectiveness globally [9]. The proportion of people with at least one vaccine dose in the least deprived areas (e.g., 68.3% in Europe) was 3.2 times higher than that in the most deprived areas (e.g., 21.3 in Africa) [10]. Similarly, the proportion of people that have been fully vaccinated in high-income countries was 5.2 times higher than that in low-income countries (79.5% vs. 15.2%, respectively) as of 25 April 2022 [11]. Such socioeconomic and geographic inequalities also exist within countries. A study in Italy found that residents with a high school degree had an odds ratio of 1.29 of not getting vaccinated compared with those with a university degree [12]. Similarly, a study in the UK found that Black residents, who are generally poorer, were 2.4 times more likely to be unvaccinated than their White counterparts [13].

In addition to inequalities in coverage, an analysis of inequalities in delays in vaccination (i.e., days to get the first and second dose) is also essential to inform the intensity of the delays. While previous studies have provided evidence of inequalities in the coverage of COVID-19 vaccination [12,13,14,15,16,17,18,19,20], evidence of socioeconomic and geographic inequalities in delays in vaccinations is lacking. As more and more countries introduce plans for living with COVID-19 [21], a better understanding of such inequalities will help in the efforts toward equal access to COVID-19 vaccines for all. Thus, our study aims to examine the socioeconomic and geographic disparities in terms of days to getting the first and second doses of COVID-19 vaccinations in Indonesia.

## 2. Methods

### 2.1. Study Setting

**Figure 1:**
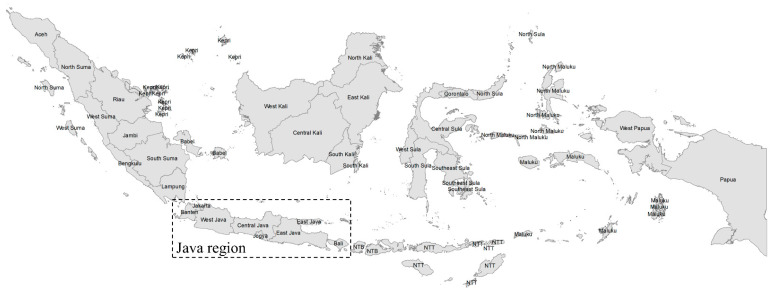
Map of Indonesia. Note: Suma = Sumatera, Kepri = Riau Islands, Sula = Sulawesi, Kali = Kalimantan, NTB = West Nusa Tenggara, NTT = East Nusa Tenggara. The Java region includes provinces in the Java and Bali islands (shown in the box). Those outside the Java region include provinces in the islands of Sumatera, Kalimantan, Sulawesi, Maluku, Nusa Tenggara, and Papua. The Java region is the most developed in the country. The authors obtained the shapefile from the Indonesian Information and Geospatial Agency and created the map in ArcMap 10.

Indonesia, a low- and middle-income country (LMIC) in the Southeast Asia region, is the fourth largest country, with a population of over 270 million. It is also an archipelago with five big islands and thousands of smaller inhabited islands (see Figure 1). Both factors contribute to more challenges in providing diagnostic, preventive, and curative health services for diseases, including during the COVID-19 pandemic. There have been over 6 million confirmed cases of COVID-19, with over 156,000 deaths reported as of 21 April 2022 [1]. Like other countries, the pandemic has also put extreme pressure on the country’s health system, which is still navigating through a major health financing reform with the introduction of a single-payer system in 2014 [22]. From January 2021, the government started mass vaccination by targeting young working people first, unlike most other countries, which began with the elderly and the most vulnerable [23]. In August 2021, the government started the booster vaccinations program, targeting the country’s 1.5 million health workers. From January 2021, as cases increased due to the Omicron variant, the government kicked off its booster drive for the general public by first prioritizing the elderly and immunocompromised residents [6]. By 21 April 2022, 198.6 million people (71.9% of the population) have received at least one vaccination, and 163.4 million people (59.1%) have received full vaccinations [11].

### 2.2. Study Design and Sample

We conducted a cross-sectional study on the geographic and socioeconomic inequalities in terms of how long (in days) it takes to receive COVID-19 vaccination in Indonesia. We collected data through the WhatsApp messaging app and other social media platforms during December 2021–February 2022. Using the Survey Monkey platform, we provided a short questionnaire link to those who agreed to be study participants. To help with the response rate, we indicated that it would take approximately five minutes in the survey invitation. Additionally, we mentioned a small incentive of IDR 5000 (~USD 0.34) to compensate for internet data usage. Our short questionnaire was adapted from the COVID-19 vaccine collaborative study conducted by the Indonesian Technical Advisory Group on Immunization (ITAGI), Ministry of Health, World Health Organization, and UNICEF [24].

In terms of sampling, the target population included five provinces, including East Java, DKI Jakarta, North Sumatra, South Sulawesi, and Papua. The minimum sample size to detect 29% vaccine coverage (i.e., national figure mid 2021) with a margin of error of 5% and a 95% confidence interval was 320 individuals, rounded off to 500 individuals due to potential non-response. By stratifying male/female, the minimum sample per province is 1000 individuals. In total, we initially targeted 5000 individuals from the five provinces.

To ensure national reach, we distributed the questionnaire link through our network of universities in all regions, including Java, Sumatra, Kalimantan, Sulawesi, Maluku, Nusa Tenggara, and Papua. We received a total of 4416 responses by the end of February 2022—an 88.3% response rate, compared to our initial target. However, because of various reasons (e.g., unwillingness to participate, missing responses, and being unvaccinated), our final analysis included 3592 participants who have received at least one dose of the vaccine and have valid answers for the month/year of receiving the first and second doses. Our inclusion criteria were males or females who are 15+ years of age and who have received at least one COVID-19 vaccination.

### 2.3. Dependent Variables

We used two main dependent variables: days to receive the first dose (after the national vaccine rollout) and days to receive the second dose (after receiving the first dose). In the survey, we asked for the month and year of receiving each dose. We did not ask for the date to reduce recall bias and help shorten the questionnaire. In creating the dependent variable, we assigned 15 as the date. We calculated the number of days to receive vaccines by subtracting each vaccine date from 13 January 2021, the start of the COVID-19 vaccine rollout in the country led by President Joko Widodo [25].

### 2.4. Independent Variables

We examined a range of socioeconomic and geographic indicators. The primary independent variables included education level, income level, formal employment, working in health facilities, being a health worker, and region. Educational levels included high school or lower, diplomas and bachelor’s degrees, and postgraduate degrees. Income levels included five groups of monthly income ranging from IDR <2 million (~USD 140) to IDR 10+ million (~USD 700). Formal employment included civil servants, government-linked company employees, private employees, and self-employed entrepreneurs. Health workers included doctors, dentists, nurses/midwives, and other health professionals. The region included those in and out of the Java region, which includes Bali, hosts the country’s national capital (Jakarta city), and is the most developed region in Indonesia. Those out of the Java region included Sumatra, Kalimantan, Sulawesi, Maluku, Nusa Tenggara, and Papua (Figure 1). We controlled for other independent variables, including sex (female/male), age group, being elderly (60+ years), religion (e.g., Islam, Hinduism, Buddhism, Christianity, Protestantism, Kong Hu Chu), and urbanicity (cities and regencies defined as urban and rural areas, respectively).

### 2.5. Data Analysis

In addition to descriptive statistics on sample characteristics, we conducted bivariate and multivariate Ordinary Least Squares (OLS) regressions in STATA 15 to examine the socioeconomic and geographic inequalities in terms of days to getting the first and second dose of COVID-19 vaccinations. For the subgroup analyses, we conducted regressions by sex (female vs. male) and region (in vs. out of Java). All statistical significances were at the 5% level or lower.

## 3. Results

Table 1 shows the descriptive statistics of the sample characteristics. In terms of characteristics (Panel A), out of the 3592 participants in our analysis, 72.6% were female. By age, the participants were grouped into 15–20 years (23.3%), 21–24 years (21.5%), 25–29 years (19.4%), 30–41 years (18.2%), and 42–76 years (17.7%). Of the participants, 2.0% were elderly (aged at least 60 years). Regarding education level, 43.1%, 44.5%, and 12.4% of the participants had a high school education or lower, diplomas or bachelor’s degrees, and postgraduate degrees, respectively. With regard to income, nearly one-third (31.2%) of the participants had an income below IDR 2 million (~USD 140), and 12.1% had an income of at least IDR 10 million (~USD 700). In terms of employment, 47.6% of participants were employed, with 18.2% and 8.6% reported as working in health and educational facilities, respectively. Of the participants, 21.6% were health workers such as doctors, dentists, nurses, and other health professionals. By region, 52.3% of the participants resided in urban areas (i.e., cities), and 56.9% lived in the Java region. Of the participants, 87.9% reported being fully vaccinated, including the first and second doses (Panel B). For the outcome variables (Panel C), the average number of days to receive the first dose was 160.7 days (about 5.4 months) from the start of the national vaccine rollout (13 January 2021). Additionally, the number of days to receive the second dose was 41.1 days after the first dose was received.

Table 2 shows bivariate analyses of socioeconomic and geographic inequalities in terms of days to receive the first and second doses of COVID-19 vaccinations. Negative values in the differences between the highest and lowest subgroups (columns 2 and 4) show fewer days to receive a vaccine (i.e., earlier). In terms of getting the first dose, on average, it took 202.6 days to receive the first dose for participants with a high school education or lower and 110.7 days for those with postgraduate degrees (columns 1–2). In terms of the difference, those with postgraduate degrees, the highest education group, received the first dose 91.9 days (about 3.1 months) earlier than those with a high school education or lower, the lowest education group. Similarly, those with the highest income level (IDR 10+ million) received the first dose 72.3 days (about 2.4 months) earlier than those with the lowest income level (IDR < 2 million). Those with formal employment, those who worked in health facilities, and health professions received the first dose 60.8 days (2.0 months), 106.3 days (3.5 months), and 97.8 days (3.3 months) earlier than others. By region, the participants who lived in the Java region received the first dose 25.2 days earlier than those out of the Java region. All these differences (inequalities) were statistically significant at the 5% level.

In terms of getting the second dose (after receiving the first dose), on average, those with the highest education and income level received the second dose 2.7 days and 0.1 days earlier than those with the lowest groups, respectively. Those with formal employment, those who worked in health facilities, and health professionals received the second dose 0.5 days, 7.6 days, and 6.1 days earlier, respectively, relative to others. Those living in Java received the second dose 4.3 days later than others. Only the differences (inequalities) in terms of working in health facilities, health professionals, and regions were statistically significant at the 5% level.

Table 3 shows the multivariate analyses of socioeconomic and geographic inequalities in terms of days to receive the first dose of COVID-19 vaccinations. Negative coefficients show fewer days to receive vaccinations by comparing each variable’s highest and lowest categories. The inequalities in terms of days to receive the first dose were relatively lower after controlling for all independent variables, but they were still statistically significant. Participants in the highest education and income groups received the first dose 30.1 days and 24.4 days earlier than those in the lowest groups, respectively. Those with formal employment, those who worked in health facilities, and health professionals received the first dose 21.1 days, 50.7 days, and 33.4 days earlier, respectively, relative to others. Those living in Java received the first dose 14.7 days earlier than others. All these differences (inequalities) were statistically significant at the 5% level.

By sex, the inequalities in terms of days to receive the first dose were relatively larger among females for all socioeconomic and geographic indicators, except for being a health professional. For instance, female and male participants in the highest education group received the first dose 33.1 and 24.2 days earlier, respectively, relative to those in the lowest education group. Female and male participants in the highest income group received the first dose 27.1 and 24.3 days earlier, respectively, relative to those in the lowest income group. Female and male participants working in health facilities received the first dose 57.4 and 35.6 days earlier, respectively, relative to others. Female and male participants living in Java received the first dose 16.3 and 11.5 days earlier, respectively, relative to others. In contrast, female and male health workers received the first dose 31.4 and 36.6 days earlier, respectively, relative to others.

By region, the patterning is mixed, with relatively larger inequalities in Java for some indicators (e.g., education, formal employment, and health workers) and smaller inequalities for others (e.g., income, worked in health facilities). Participants in the highest education group in and out of Java received the first dose 36.7 and 20.7 days earlier, respectively, relative to those in the lowest education group. In and out of Java, those with formal employment received the first dose 25.6 and 14.2 days earlier, respectively, relative to others. In and out of Java, those in health professions received the first dose 38.3 and 25.3 days earlier, respectively, relative to others. In contrast, those in the highest income group in and out of Java received the first dose 22.3 and 28.3 days earlier, respectively, relative to those in the lowest income group.

Table 4 shows the multivariate analyses of socioeconomic and geographic inequalities in terms of days to receive the second dose of COVID-19 vaccinations. Negative/positive coefficients show fewer/more days to receive vaccinations by comparing each variable’s highest and lowest categories. The inequalities in terms of days to receive the second dose were not statistically significant in terms of education, income, formal employment, and health professions. However, those who worked in health facilities received the second dose 7.9 days earlier and those living in Java received the second dose 4.9 days later compared to others (statistically significant at the 5% level). By subgroup, the inequalities in terms of working in health facilities were larger among females (9.7 and 4.3 days earlier for female and male participants, respectively) and those out of the Java region (5.3 and 12.6 days earlier for in and out of Java, respectively). Additionally, the inequalities by region were slightly larger among males (4.6 and 5.5 days earlier for female and male participants, respectively).

## 4. Discussion

Our findings show considerable delays in getting the first dose among participants (160.7 days or about 5.4 months on average) from the start of Indonesia’s national COVID-19 vaccination rollout on 13 January 2021. However, we found a shorter period to receive the second dose after receiving the first dose (41.1 days on average). Moreover, we found significant socioeconomic (i.e., education, income, formal employment, working in health facilities, and being a health worker) and geographic (i.e., in and out of the Java region) inequalities in terms of delays in getting the first dose. However, we did not find significant inequalities in getting the second dose for most inequality indicators, except for working in health facilities. By region, we even found that participants living out of the Java region received the second dose 4.9 days earlier.

We found that participants in the highest education and income groups received the first dose 30.1 days and 24.4 days earlier than those in the lowest groups. Moreover, those with formal employment received the first dose 21.1 days earlier than those without formal employment. To our knowledge, there are currently no previous studies to compare. However, our findings align with previous studies on the inequalities in COVID-19 vaccination coverage. For instance, a study in Italy found that those in the lowest education group had an odds ratio of 1.29 of not getting vaccinated compared with those with a university degree [12]. Additionally, another study in the UK showed that Black residents, who were generally poorer, were 2.4 times more likely to be unvaccinated [13]. In terms of the comparison of the delays in vaccination, our findings align with previous studies on the inequalities in child vaccination delays. A study in the UK found that routine child vaccination delay was pronounced for the 40% most deprived population [26]. Another study from sub-Saharan Africa found that inequalities in vaccination delay in terms of household wealth, place of residence, and education existed in most countries [27].

Our findings found that those working in health facilities and professions received the first dose 50.7 days and 33.4 days earlier, respectively, relative to others. This was due to the national policy to prioritize those working in health facilities and health workers being among the first group to receive COVID-19 vaccinations. This was in line with the World Health Organization’s recommendation to prioritize groups at the highest risk of exposure to infection in each country, including health workers [28].

However, in terms of getting the second dose, we did not find significant inequalities for most inequality indicators. This means that while there were considerable delays in getting the first dose, especially if someone was of a lower socioeconomic status or lived in more deprived areas, the waiting time for the second dose was relatively similar for everyone once they were in the system. Moreover, we also found that participants living in the more deprived areas (i.e., out of the Java region) received the second dose earlier. This may be due to the large number of people that needed to be vaccinated in the Java region (including Bali province), where over half of the country’s population lives.

There are several factors that may contribute to the geographic and socioeconomic inequalities in terms of delays in getting the COVID-19 vaccine: vaccine unavailability, a lack of information, and vaccine hesitancy. In terms of unavailability, the government reported in August 2021 that the areas with the least availability were all outside of Java/Bali, including Sumatera, Kalimantan, and the Maluku and Papua regions [29]. In terms of the lack of information, a study in Hong Kong found that Chinese adults with a higher socioeconomic status had higher eHealth literacy and sought more web-based information on COVID-19. The two factors were found to be associated with a high adherence to the guidelines for preventive behaviors during the pandemic [30]. In terms of hesitancy, the evidence is mixed. A global study of 20 countries (including Indonesia) in early 2021 showed that hesitancy towards COVID-19 vaccines was associated with higher education, being employed, and lower income [31]. Moreover, political persuasion may impact vaccination penetration, but the time between vaccination is less impacted by politics.

For policy, our findings provide evidence for the government and policymakers in Indonesia and other LMICs with similar settings to reduce socioeconomic and geographic inequalities in delays of COVID-19 vaccinations. All LMICs struggled to implement successful mass COVID-19 vaccination programs during the pandemic. Given the archipelagic setting, the government and policymakers have additional challenges with the huge population distributed unequally in all regions, including thousands of inhabited islands. Additionally, since comprehensive vaccination against COVID-19 could reduce these inequalities arising out of the pandemic, further efforts to reach lower socioeconomic groups are essential [12].

The government in Indonesia and other LMICs need to make various efforts to increase complete vaccination coverage for all (universal), especially among vulnerable groups such as the elderly, those with medical conditions associated with a higher risk for severe COVID-19, and health workers. They also need to ensure the equal distribution of vaccination coverage to all groups, especially those from low socioeconomic groups (pro-poor). In the United Kingdom, for example, NHS England provided additional financial support to increase the uptake of COVID-19 vaccines among ethnic minorities and low socioeconomic groups [32]. In addition, local government agencies and local community organizations helped through training and health promotion to ensure that messages reached the community, helpline numbers, working with youth groups, and social media campaigns to fight anti-vaccination campaigns [33]. In the United States, activities included building trust, conducting outreach, and working with influential religious organizations in certain community groups [33].

Our study has at least two limitations. First, given the restrictions on movement and gathering during the pandemic, our survey was conducted online (web-based), which may have an inherent bias with respect to socioeconomic factors and limits the population representativeness of our findings [34,35,36,37]. Compared to the population census 2020, some characteristics of our study participants are similar (e.g., proportion of participants in the Java region), but some are not (e.g., our sample had a higher proportion of females and a lower proportion of older adults) [38]. Second, because a cross-sectional study design was employed, our results show associations, not causality. Regardless of these limitations, our findings have important policy implications for Indonesia and other LMICs with similar settings.

## 5. Conclusions

In Indonesia, we found considerable delays in getting the first dose among the study participants—160.7 days, on average, from the national COVID-19 vaccination rollout on 13 January 2021. We found a shorter period to receive the second dose—41.1 days, on average, from receiving the first dose. We found significant socioeconomic (i.e., education, income, formal employment, working in health facilities, and being a health worker) and geographic (i.e., in and out of the Java region) inequalities in terms of delays in getting the first dose. However, we did not find significant inequalities in getting the second dose for most inequality indicators, except for working in health facilities. By region, we found that participants living in more deprived areas (out of the Java region) received the second dose 4.9 days earlier. Effective efforts to address inequalities are essential to ensuring the effectiveness of the national COVID-19 vaccination rollout.

## Figures and Tables

**Table 1:** Sample characteristics.

	n	%
(a) Basic characteristics (n, %)		
Female	2609	72.6%
Age		
15–20 years	836	23.3%
21–24 years	772	21.5%
25–29 years	695	19.4%
30–41 years	653	18.2%
42–76 years	636	17.7%
Elderly	70	2.0%
Education		
High school or lower	1548	43.1%
Diploma or bachelor’s degree	1599	44.5%
Postgraduate degree	445	12.4%
Monthly income		
IDR < 2 million	1121	31.2%
2–4 million	860	23.9%
4–6 million	640	17.8%
6–10 million	536	14.9%
10+ million	435	12.1%
Employed	1708	47.6%
Work in health facilities	653	18.2%
Work in educational facilities	310	8.6%
Health workers	776	21.6%
Married	1561	43.5%
Muslim	3246	90.4%
Urban area	1877	52.3%
Java region	2043	56.9%
(b) Vaccination coverage (n, %)		
Had COVID-19 vaccine: 1 dose	3592	100.0%
Had COVID-19 vaccine: 2 doses	3157	87.9%
(c) Outcome variables (mean, SD)		
Days to receive the first dose (after the vaccine rollout started)	160.7	87.8
Days to receive the second dose (after the first dose was received)	41.1	37.9
N, %	3592	

Note: N/n = sample, IDR = Indonesian Rupiah. Elderly = 60+ years old (local definition). Employed includes civil servants, government-linked company employees, private employees, and self-employed entrepreneurs. Health workers include doctors, dentists, nurses/midwives, and other health professionals. Muslims include those who follow Islam; other religions include Hinduism, Buddhism, Christianity, Protestantism, and Kong Hu Chu. Urban areas include cities; rural areas include regencies/districts. The Java region includes Bali; other regions include Sumatra, Kalimantan, Sulawesi, Maluku, Nusa Tenggara, and Papua.

**Table 2:** Bivariate analysis of inequalities in days to receive first and second doses of the COVID-19 vaccine.

	Days to Receive First Dose		Days to Receive Second Dose	
		Difference		Difference
	Mean	(High-Low)	Mean	(High-Low)
	(1)	(2)	(3)	(4)
Education				
High school or lower	202.6	−91.9 *	43.1	−2.7
Diploma and bachelor’s degree	134.0		39.4	
Postgraduate degree	110.7		40.4	
Monthly income				
IDR < 2 million	192.4	−72.3 *	42.0	−0.1
2–4 million	159.1		41.7	
4–6 million	149.4		41.2	
6–10 million	143.4		37.2	
10+ million	120.1		41.9	
Employed				
No	185.7	−60.8 *	41.3	−0.5
Yes	124.9		40.8	
Work in health facilities				
No	180.0	−106.3 *	42.5	−7.6 *
Yes	73.7		34.9	
Health workers				
No	181.8	−97.8 *	42.4	−6.1 *
Yes	84.0		36.3	
Region				
Outside Java	175.0	−25.2 *	38.6	4.3 *
Java	149.8		42.9	

Note: Diff = Difference, High = Highest, Low = Lowest, IDR = Indonesian Rupiah. Negative values show a fewer number of days to receive the vaccine. Formally employed includes civil servants, government-linked company employees, and private employees. Health workers include doctors, dentists, nurses/midwives, and other health professionals. The Java region includes Bali; other regions include Sumatra, Kalimantan, Sulawesi, Maluku, Nusa Tenggara, and Papua. * = statistical significance level * *p* < 0.05 estimated in bivariate Ordinary Least Squares regressions in STATA 15.

**Table 3:** Socioeconomic and geographic inequalities in days to receive the COVID-19 vaccine: first dose.

	Total		Female		Male		Java Region		Outside Java	
Variables	Coef	95%CI	Coef	95%CI	Coef	95%CI	Coef	95%CI	Coef	95%CI
	(1)		(2)		(3)		(4)		(5)	
Female	1.9	(−3.5, 7.4)					−1.3	(−8.3, 5.7)	7.0	(−1.7, 15.7)
Age										
15–20 years										
21–24 years	3.1	(−4.6, 10.9)	2.7	(−5.9, 11.2)	4.0	(−14.0, 22.1)	−1.7	(−12.5, 9.1)	6.2	(−5.2, 17.6)
25–29 years	−8.8	(−18.6, 0.9)	−9.1	(−20.1, 1.8)	−6.4	(−28.3, 15.6)	−10.7	(−23.7, 2.2)	−9.1	(−24.7, 6.4)
30–41 years	−20.9 *	(−32.3, −9.5)	−23.1 *	(−35.8, −10.3)	−5.2	(−31.4, 20.9)	−30.5 *	(−45.0, −16.0)	−7.6	(−26.8, 11.6)
42–76 years	−40.6 *	(−52.6, −28.5)	−41.2 *	(−55.1, −27.4)	−24.3	(−50.6, 1.9)	−47.0 *	(−61.9, −32.1)	−31.7 *	(−53.0, −10.3)
Elderly	−10.6	(−28.4, 7.2)	−19.6	(−53.4, 14.1)	−9.3	(−32.6, 14.0)	−9.6	(−29.0, 9.8)	−15.8	(−57.8, 26.2)
Education										
High school/less										
Diploma/bachelor	−24.8 *	(−31.7, −17.8)	−25.4 *	(−33.5, −17.3)	−20.7 *	(−34.5, −6.8)	−28.4 *	(−37.1, −19.7)	−18.8 *	(−30.6, −7.1)
Postgraduate	−30.1 *	(−40.1, −20.2)	−33.1 *	(−44.9, −21.2)	−24.2 *	(−42.9, −5.6)	−36.7 *	(−49.0, −24.4)	−20.7 *	(−37.7, −3.6)
Income in IDR										
<2 million										
2–4 million	−5.6	(−12.2, 0.9)	−3.0	(−10.4, 4.4)	−14.5 *	(−28.7, −0.3)	−7.1	(−15.8, 1.7)	−4.6	(−14.6, 5.5)
4–6 million	−9.2 *	(−16.6, −1.9)	−7.8	(−16.1, 0.5)	−16.0 *	(−31.7, −0.3)	−9.0	(−18.5, 0.6)	−9.4	(−21.1, 2.2)
6–10 million	−11.3 *	(−19.2, −3.4)	−11.7 *	(−20.8, −2.7)	−13.8	(−29.9, 2.3)	−10.2	(−20.7, 0.3)	−14.3 *	(−26.4, −2.2)
10+ million	−24.4 *	(−33.2, −15.7)	−27.1 *	(−37.2, −17.0)	−24.3 *	(−42.0, −6.7)	−22.3 *	(−32.9, −11.8)	−28.3 *	(−44.7, −12.0)
Formally employed	−21.1 *	(−27.4, −14.7)	−21.6 *	(−29.1, −14.0)	−16.1 *	(−28.3, −3.9)	−25.6 *	(−33.4, −17.8)	−14.2 *	(−25.1, −3.3)
Work in health facilities	−50.7 *	(−60.1, −41.3)	−57.4 *	(−68.3, −46.5)	−35.6 *	(−54.5, −16.6)	−38.4 *	(−49.7, −27.0)	−71.2 *	(−87.7, −54.7)
Work in educ facilities	6.1	(−3.6, 15.9)	5.6	(−5.6, 16.8)	4.5	(−15.6, 24.6)	8.7	(−4.2, 21.6)	1.2	(−13.9, 16.3)
Health workers	−33.4 *	(−42.4, −24.5)	−31.4 *	(−41.8, −21.1)	−36.6 *	(−54.6, −18.5)	−38.3 *	(−49.5, −27.2)	−25.3 *	(−40.4, −10.2)
Married	21.0 *	(13.7, 28.2)	26.7 *	(18.8, 34.7)	−4.3	(−22.2, 13.6)	24.7 *	(15.8, 33.7)	12.8 *	(0.4, 25.2)
Muslim	12.9 *	(5.0, 20.9)	15.0 *	(5.3, 24.6)	9.5	(−5.0, 24.0)	9.8	(−0.9, 20.6)	16.4 *	(4.4, 28.4)
Urban	−3.1	(−7.9, 1.7)	−2.9	(−8.5, 2.6)	−2.4	(−12.2, 7.4)	−6.7 *	(−12.9, −0.4)	−1.3	(−9.0, 6.4)
Java region	−14.7 *	(−19.6, −9.8)	−16.3 *	(−21.8, −10.7)	−11.5 *	(−21.8, −1.2)				
Constant	207.6 *	(197.1, 218.2)	208.1 *	(196.8, 219.4)	210.3 *	(189.8, 230.8)	205.3 *	(190.8, 219.8)	195.0 *	(179.5, 210.5)
N	3592		2609		983		2043		1549	

Note: N = sample, IDR = Indonesian Rupiah, Coef = Coefficients (negative values show a fewer number of days to receive the vaccine), CI = Confidence interval (shown in parentheses), NA = Not Applicable, Ref = Reference. Elderly = 60+ years old. Formally employed includes civil servants, government-linked company employees, and private employees. Health workers include doctors, dentists, nurses/midwives, and other health professionals. Muslims include those who follow Islam; other religions include Hinduism, Buddhism, Christianity, Protestantism, and Kong Hu Chu. Urban areas include cities; rural areas include regencies/districts. The Java region includes Bali; other regions include Sumatra, Kalimantan, Sulawesi, Maluku, Nusa Tenggara, and Papua. Significance level * *p* < 0.05.

**Table 4:** Socioeconomic and geographic inequalities in days to receive the COVID-19 vaccine: second dose.

	Total		Female		Male		Java Region		Outside Java	
Variables	Coef	95%CI	Coef	95%CI	Coef	95%CI	Coef	95%CI	Coef	95%CI
	(1)		(2)		(3)		(4)		(5)	
Female	−0.7	(−3.4, 2.0)					−1.4	(−5.1, 2.2)	0.3	(−3.8, 4.4)
Age										
15–20 years										
21–24 years	0.1	(−3.8, 4.0)	1.7	(−2.5, 6.0)	−5.3	(−14.5, 3.9)	1.3	(−4.3, 6.9)	0.5	(−5.1, 6.0)
25–29 years	−3.1	(−8.0, 1.9)	−2.5	(−8.0, 3.0)	−4.8	(−16.1, 6.5)	0.9	(−5.9, 7.6)	−5.7	(−13.2, 1.9)
30–41 years	1.2	(−4.5, 7.0)	2.6	(−3.7, 9.0)	−1.2	(−14.8, 12.4)	5.6	(−2.0, 13.2)	−3.1	(−12.4, 6.1)
42–76 years	2.6	(−3.5, 8.7)	4.7	(−2.3, 11.6)	−0.6	(−14.2, 12.9)	5.1	(−2.7, 12.9)	1.7	(−8.6, 12.0)
Elderly	−3.9	(−13.0, 5.2)	−14.2	(−31.9, 3.5)	−1.5	(−13.4, 10.5)	−0.0	(−10.3, 10.3)	−18.6	(−39.4, 2.1)
Education										
High school/less										
Diploma/bachelor	−1.1	(−4.7, 2.5)	−1.7	(−5.8, 2.4)	0.0	(−7.3, 7.3)	−0.3	(−4.9, 4.3)	−2.1	(−7.8, 3.6)
Postgraduate	−1.5	(−6.4, 3.5)	−3.2	(−9.1, 2.6)	0.3	(−9.4, 10.1)	−4.4	(−10.8, 2.0)	3.4	(−4.8, 11.5)
Income in IDR										
<2 million										
2–4 million	0.6	(−2.7, 3.9)	0.1	(−3.6, 3.8)	2.4	(−4.9, 9.7)	−1.1	(−5.6, 3.4)	2.5	(−2.3, 7.3)
4–6 million	−0.8	(−4.4, 2.9)	0.3	(−3.7, 4.4)	−3.8	(−11.9, 4.3)	−2.4	(−7.3, 2.6)	1.0	(−4.4, 6.4)
6–10 million	−4.6 *	(−8.6, −0.7)	−7.1 *	(−11.6, −2.6)	0.7	(−7.4, 8.8)	−5.2	(−10.6, 0.2)	−4.8	(−10.5, 0.9)
10+ million	−1.0	(−5.3, 3.3)	−3.9	(−8.8, 1.0)	4.8	(−4.0, 13.7)	−0.1	(−5.5, 5.3)	−4.9	(−12.5, 2.7)
Formally employed	2.6	(−0.6, 5.7)	2.5	(−1.2, 6.2)	4.8	(−1.4, 11.0)	0.9	(−3.1, 4.9)	5.3 *	(0.1, 10.4)
Work in health facilities	−7.9 *	(−12.5, −3.3)	−9.7 *	(−14.9, −4.5)	−4.3	(−14.1, 5.4)	−5.3	(−11.1, 0.5)	−12.6 *	(−20.4, −4.9)
Work in educ facilities	−1.3	(−6.1, 3.5)	−4.4	(−9.8, 1.1)	7.0	(−3.1, 17.1)	−1.8	(−8.4, 4.8)	−1.5	(−8.7, 5.6)
Health workers	−0.4	(−4.8, 4.0)	0.7	(−4.3, 5.7)	−3.6	(−12.9, 5.7)	−1.3	(−7.0, 4.4)	0.5	(−6.6, 7.6)
Married	−1.8	(−5.5, 1.8)	−1.4	(−5.4, 2.5)	−3.8	(−13.1, 5.6)	−4.1	(−8.8, 0.6)	1.4	(−4.5, 7.3)
Muslim	−5.5 *	(−9.4, −1.5)	−3.0	(−7.7, 1.7)	−10.8 *	(−18.1, −3.4)	−9.5 *	(−15.0, −3.9)	−1.4	(−7.1, 4.2)
Urban	0.1	(−2.3, 2.5)	1.4	(−1.4, 4.1)	−3.3	(−8.3, 1.6)	1.6	(−1.6, 4.8)	−3.0	(−6.6, 0.7)
Java region	4.9 *	(2.5, 7.4)	4.6 *	(1.9, 7.3)	5.5 *	(0.3, 10.7)				
Constant	46.5 *	(41.2, 51.8)	43.4 *	(37.8, 49.0)	52.0 *	(41.6, 62.4)	54.6 *	(47.1, 62.0)	43.2 *	(35.8, 50.6)
N	3102		2261		841		1794		1308	

Note: N = sample, IDR = Indonesian Rupiah, Coef = Coefficients (negative values show a fewer number of days to receive the vaccine), CI = Confidence interval (shown in parentheses), NA = Not Applicable, Ref = Reference. Elderly = 60+ years old. Formally employed includes civil servants, government-linked company employees, and private employees. Health workers include doctors, dentists, nurses/midwives, and other health professionals. Muslims include those who follow Islam; other religions include Hinduism, Buddhism, Christianity, Protestantism, and Kong Hu Chu. Urban areas include cities; rural areas include regencies/districts. The Java region includes Bali; other regions include Sumatra, Kalimantan, Sulawesi, Maluku, Nusa Tenggara, and Papua. Values were estimated in Ordinary Least Squares regression in STATA 15. Statistical significance level * *p* < 0.05.

## Data Availability

Available from the authors upon reasonable request.

## References

[1] WHO (2022). WHO Coronavirus (COVID-19) Dashboard. https://covid19.who.int/.

[2] Oxford (2022). COVID-19 Government Response Tracker|Blavatnik School of Government. https://www.bsg.ox.ac.uk/research/research-projects/covid-19-government-response-tracker.

[3] CNN (2022). Travel Restrictions by Country Following the Omicron Variant Outbreak|CNN Travel. https://edition.cnn.com/travel/article/coronavirus-omicron-variant-travel-restrictions/index.html.

[4] UK Government One Year Anniversary of UK Deploying Oxford-AstraZeneca Vaccine—GOV.UK. https://www.gov.uk/government/news/one-year-anniversary-of-uk-deploying-oxford-astrazeneca-vaccine.

[5] BBC COVID-19 Vaccine: First Person Receives Pfizer Jab in UK—BBC News. https://www.bbc.co.uk/news/uk-55227325.

[6] Reuters Indonesia Rolls out Booster Shots, Amid Fears of Omicron Spread|Reuters. https://www.reuters.com/business/healthcare-pharmaceuticals/indonesia-rolls-out-booster-shots-amid-fears-omicron-spread-2022-01-12/.

[7] Abu-Raddad L.J., Chemaitelly H., Butt A.A. (2021). Effectiveness of the BNT162b2 COVID-19 Vaccine against the B.1.1.7 and B.1.351 Variants. N. Engl. J. Med..

[8] BBC COVID Vaccines—‘Spectacular’ Impact on Serious Illness—BBC News. https://www.bbc.co.uk/news/health-56153600.

[9] ECDC SARS-CoV-2 Variants of Concern as of 11 March 2022. https://www.ecdc.europa.eu/en/covid-19/variants-concern.

[10] Holder J. (2022). COVID World Vaccination Tracker—The New York Times. https://www.nytimes.com/interactive/2021/world/covid-vaccinations-tracker.html.

[11] Our World in Data Coronavirus (COVID-19) Vaccinations—Our World in Data. https://ourworldindata.org/covid-vaccinations.

[12] Cesaroni G., Calandrini E., Balducci M., Cappai G., Di Martino M., Sorge C., Nicastri E., Agabiti N., Davoli M. (2022). Educational Inequalities in COVID-19 Vaccination: A Cross-Sectional Study of the Adult Population in the Lazio Region, Italy. Vaccines.

[13] Watkinson R.E., Williams R., Gillibrand S., Sanders C., Sutton M. (2022). Ethnic inequalities in COVID-19 vaccine uptake and comparison to seasonal influenza vaccine uptake in Greater Manchester, UK: A cohort study. PLoS Med..

[14] Perry M., Akbari A., Cottrell S., Gravenor M.B., Roberts R., Lyons R.A., Bedston S., Torabi F., Griffiths L. (2021). Inequalities in coverage of COVID-19 vaccination: A population register based cross-sectional study in Wales, UK. Vaccine.

[15] Curtis H.J., Inglesby P., Morton C.E., MacKenna B., Green A., Hulme W., Walker A.J., Morley J., Mehrkar A., Bacon S. (2022). Trends and clinical characteristics of COVID-19 vaccine recipients: A federated analysis of 57.9 million patients’ primary care records in situ using OpenSAFELY. Br. J. Gen. Pract..

[16] Nafilyan V., Dolby T., Finning K., Morgan J., Edge R., Glickman M., Pearce N., van Tongeren M. (2022). Differences in COVID-19 vaccination coverage by occupation in England: A national linked data study. Occup. Environ. Med..

[17] Williams A.M., Clayton H.B., Singleton J.A. (2022). Racial and Ethnic Disparities in COVID-19 Vaccination Coverage: The Contribution of Socioeconomic and Demographic Factors. Am. J. Prev. Med..

[18] Spetz M., Lundberg L., Nwaru C., Li H., Santosa A., Leach S., Gisslén M., Hammar N., Rosvall M., Nyberg F. (2022). The social patterning of COVID-19 vaccine uptake in older adults: A register-based cross-sectional study in Sweden. Lancet Reg. Health Eur..

[19] Saban M., Myers V., Ben-Shetrit S., Wilf-Miron R. (2021). Socioeconomic gradient in COVID-19 vaccination: Evidence from Israel. Int. J. Equity Health.

[20] Oroszi B., Juhász A., Nagy C., Horváth J.K., Komlós K.E., Túri G., McKee M., Ádány R. (2022). Characteristics of the Third COVID-19 Pandemic Wave with Special Focus on Socioeconomic Inequalities in Morbidity, Mortality and the Uptake of COVID-19 Vaccination in Hungary. J. Pers. Med..

[21] Smith-Spark L. (2021). Living with COVID: Five Countries That Have Decided It’s Time to Open Up—CNN. https://edition.cnn.com/2021/09/16/world/covid-countries-opening-up-cmd-intl/index.html.

[22] Kusuma D., Ahsan A. (2022). Political economy of Universal Health Coverage in Indonesia. Introduction to Health Economics.

[23] Henschke R., Anugrah P. Indonesia Coronavirus: The Vaccination Drive Targeting Younger People—BBC News. https://www.bbc.co.uk/news/world-asia-55620356.

[24] ITAGI (2020). COVID-19 Vaccine Acceptance Survey in Indonesia.

[25] MOH (2021). Program Vaksinasi COVID-19 Mulai Dilakukan, Presiden Orang Pertama Penerima Suntikan Vaksin COVID-19—P2P Kemenkes RI. http://p2p.kemkes.go.id/program-vaksinasi-covid-19-mulai-dilakukan-presiden-orang-pertama-penerima-suntikan-vaksin-covid-19/.

[26] Haider E.A., Willocks L.J., Anderson N. (2019). Identifying inequalities in childhood immunisation uptake and timeliness in southeast Scotland, 2008–2018: A retrospective cohort study. Vaccine.

[27] Mutua M.K., Mohamed S.F., Porth J.M., Faye C.M. (2021). Inequities in On-Time Childhood Vaccination: Evidence from Sub-Saharan Africa. Am. J. Prev. Med..

[28] WHO COVID-19 Vaccines. https://www.who.int/westernpacific/emergencies/covid-19/covid-19-vaccines.

[29] Satryo A. (2021). Kesenjangan Vaksin Terjadi di Luar Jawa-Bali, Masyarakat Sipil Minta Pemerintah Lebih Giat Jemput Bola. https://politik.rmol.id/read/2021/08/26/501963/kesenjangan-vaksin-terjadi-di-luar-jawa-bali-masyarakat-sipil-minta-pemerintah-lebih-giat-jemput-bola.

[30] Guo Z., Zhao S.Z., Guo N., Wu Y., Weng X., Wong J.Y.-H., Lam T.H., Wang M.P. (2021). Socioeconomic Disparities in eHealth Literacy and Preventive Behaviors During the COVID-19 Pandemic in Hong Kong: Cross-sectional Study. J. Med. Internet Res..

[31] Marzo R.R., Ahmadnd A., Islam M.S., Essar M.Y., Heidler P., King I., Thiyagarajan A., Jermsittiparsert K., Songwathana K., Younus D.A. (2022). Perceived COVID-19 vaccine effectiveness, acceptance, and drivers of vaccination decision-making among the general adult population: A global survey of 20 countries. PLoS Negl. Trop. Dis..

[32] Iacobucci G. (2021). COVID-19: NHS England pledges extra funding to local areas to reduce vaccine inequalities. BMJ.

[33] Wilkinson E. (2021). COVID-19 vaccine outreach: “local knowledge, contacts, and credibility really, really matter”. BMJ.

[34] Bradley V.C., Kuriwaki S., Isakov M., Sejdinovic D., Meng X.-L., Flaxman S. (2021). Unrepresentative big surveys significantly overestimated US vaccine uptake. Nature.

[35] Drobniewski F., Kusuma D., Broda A., Castro-Sánchez E., Ahmad R. (2022). COVID-19 Vaccine Hesitancy in Diverse Groups in the UK—Is the Driver Economic or Cultural in Student Populations. Vaccines.

[36] Bella A., Akbar M., Kusnadi G., Herlinda O., Regita P., Kusuma D. (2021). Socioeconomic and Behavioral Correlates of COVID-19 Infections among Hospital Workers in the Greater Jakarta Area, Indonesia: A Cross-Sectional Study. Int. J. Environ. Res. Public Health.

[37] Wulan W.R., Kusuma D., Nurjanah N., Aprianti A., Ahsan A. (2022). Is Exposure to Social Media Advertising and Promotion Associated with E-cigarette Use? Evidence from Indonesia. Asia Pac. J. Cancer Prev..

[38] Statistics Bureau and Ministry of Home Affairs Results of Population Census 2020. https://www.bps.go.id/pressrelease/2021/01/21/1854/hasil-sensus-penduduk-2020.html.

